# A mixed method multi-country assessment of barriers to implementing pediatric inpatient care guidelines

**DOI:** 10.1371/journal.pone.0212395

**Published:** 2019-03-25

**Authors:** Kirkby D. Tickell, Dorothy I. Mangale, Stephanie N. Tornberg-Belanger, Celine Bourdon, Johnstone Thitiri, Molline Timbwa, Jenala Njirammadzi, Wieger Voskuijl, Mohammod J. Chisti, Tahmeed Ahmed, Abu S. M. S. B. Shahid, Abdoulaye H. Diallo, Issaka Ouédrago, Al Fazal Khan, Ali F. Saleem, Fehmina Arif, Zaubina Kazi, Ezekiel Mupere, John Mukisa, Priya Sukhtankar, James A. Berkley, Judd L. Walson, Donna M. Denno, on behalf of the Childhood Acute Illness and Nutrition Network

**Affiliations:** 1 Department of Epidemiology, University of Washington, Seattle, Washington, United States of America; 2 Department of Global Health, University of Washington, Seattle, Washington, United States of America; 3 Program in Translational Medicine, The Hospital for Sick Children, Toronto, Ontario, Canada; 4 KEMRI-Wellcome Trust Research Programe, Kilifi, Kenya; 5 Department of Paediatrics, College of Medicine, University of Malawi, Blantyre, Malawi; 6 Global Child Health Group, Emma Children’s Hospital, Amsterdam University Medical Centre, Amsterdam, the Netherlands; 7 Centre for Nutrition & Food Security (CNFS), icddr, b, Dhaka, Bangladesh; 8 Department of Public Health, Centre MURAZ Research Institute, Ministry of Health, Bobo-Dioulasso, Burkina Faso; 9 Department of Public Health, University of Ouagadougou, Ouagadougou, Burkina Faso; 10 Department of Paediatrics, Banfora Regional Referral Hospital, Banfora, Burkina Faso; 11 Department of Paediatrics and Child Health, Aga Khan University, Karachi, Pakistan; 12 Department of Paediatrics, Civil Hospital Karachi, Karachi, Pakistan; 13 Department of Paediatrics and Child Health, College of Health Sciences, Makerere University, Kampala, Uganda; 14 Department of Medicine, University of Washington, Seattle, Washington, United States of America; 15 Department of Pediatrics, University of Washington, Seattle, Washington, United States of America; 16 Department of Health Services, University of Washington, Seattle, Washington, United States of America; Federal University of Sergipe, BRAZIL

## Abstract

**Introduction:**

Accelerating progress in reducing child deaths is needed in order to achieve the Sustainable Development Goal child mortality target. This will require a focus on vulnerable children–including young children, those who are undernourished or with acute illnesses requiring hospitalization. Improving adherence to inpatient guidelines may be an important strategy to reduce child mortality, including among the most vulnerable. The aim of our assessment of nine sub-Saharan African and South Asian hospitals was to determine adherence to pediatric inpatient care recommendations, in addition to capacity for and barriers to implementation of guideline-adherent care prior to commencing the Childhood Acute Illness and Nutrition (CHAIN) Cohort study. The CHAIN Cohort study aims to identify modifiable risk factors for poor inpatient and post discharge outcomes above and beyond implementation of guidelines.

**Methods:**

Hospital infrastructure, staffing, durable equipment, and consumable supplies such as medicines and laboratory reagents, were evaluated through observation and key informant interviews. Inpatient medical records of 2–23 month old children were assessed for adherence to national and international guidelines. The records of children with severe acute malnutrition (SAM) were oversampled to reflect the CHAIN study population. Seven core adherence indicators were examined: oximetry and oxygen therapy, fluids, anemia diagnosis and transfusion, antibiotics, malaria testing and antimalarials, nutritional assessment and management, and HIV testing.

**Results:**

All sites had facilities and equipment necessary to implement care consistent with World Health Organization and national guidelines. However, stockouts of essential medicines and laboratory reagents were reported to be common at some sites, even though they were mostly present during the assessment visits. Doctor and nurse to patient ratios varied widely. We reviewed the notes of 261 children with admission diagnoses of sepsis (17), malaria (47), pneumonia (70), diarrhea (106), and SAM (119); 115 had multiple diagnoses. Adherence to oxygen therapy, antimalarial, and malnutrition refeeding guidelines was >75%. Appropriate antimicrobials were prescribed for 75% of antibiotic-indicative conditions. However, 20/23 (87%) diarrhea and 20/27 (74%) malaria cases without a documented indication were prescribed antibiotics. Only 23/122 (19%) with hemoglobin levels meeting anemia criteria had recorded anemia diagnoses. HIV test results were infrequently documented even at hospitals with universal screening policies (66/173, 38%). Informants at all sites attributed inconsistent guideline implementation to inadequate staffing.

**Conclusion:**

Assessed hospitals had the infrastructure and equipment to implement guideline-consistent care. While fluids, appropriate antimalarials and antibiotics, and malnutrition refeeding adherence was comparable to published estimates from low- and high-resource settings, there were inconsistencies in implementation of some other recommendations. Stockouts of essential therapeutics and laboratory reagents were a noted barrier, but facility staff perceived inadequate human resources as the primary constraint to consistent guideline implementation.

## Introduction

Global child mortality rates remain unacceptably high at 39 deaths per 1000 live births, despite reductions over the past decades.[1] The vast majority of the more than five million under-5 deaths per annum occur in low-resource settings. To achieve the Sustainable Development Goals child health target, accelerated progress is required which will necessitate focusing on children at highest risk of death.[2] Standardized case management of acutely ill children requiring hospitalization, based on World Health Organization (WHO) guidelines, has been shown to reduce inpatient mortality, and many avoidable child deaths can be attributed to a failure to administer recommended care.[3–7] However, hospitals implementing these guidelines still report high inpatient and post-discharge case fatality rates.[3,8–10]

The objective of the Childhood Acute Illness & Nutrition (CHAIN) Network is to build the evidence base for improving outcomes of children most vulnerable to poor outcomes. These vulnerabilities include young age, undernutrition, and acute illness warranting hospitalization. By recruiting a cohort of 2–23 month-old children at hospital admission and following them six months post-discharge, the CHAIN Network aims to identify modifiable causes of inpatient and post-discharge mortality beyond a failure to correctly implement existing guidelines. The aim of the evaluation described in this paper was to determine clinical capacity, adherence to pediatric inpatient care standards, and capacity for and barriers to implementation of guideline-adherent care at each site prior to the cohort study initiation. The nine Network sites include: Dhaka and Matlab Hospitals (Bangladesh), the Civil Hospital Karachi (Pakistan), Kilifi County Hospital (Kenya), Mbagathi District Hospital (Kenya), Migori Country Referral Hospital (Kenya), Mulago Hospital (Uganda), Queen Elizabeth Central Hospital (Malawi), and Banfora Regional Referral Hospital (Burkina Faso). This paper describes these sites, clinical care delivered prior to beginning cohort recruitment, and the barriers to guideline implementation experienced by the Network hospitals.

## Methods

We conducted a mixed method assessment of the nine CHAIN Network sites. Key health indicators (fertility, under-five mortality, malaria incidence, and HIV, child wasting and stunting prevalences) for the population served by each facility were extracted from publicly available sources in order to provide a context for the sites. Our assessment framework and standardized data collection tools (Table A in S1 File) were developed based on published quality of care appraisals and WHO quality assessment references.[11–18] At least two assessment team members (DD, DM, KT, PS, CB) visited each hospital between May 2016 and October 2017, and collected information across five domains (listed below) through direct observation, clinical notes review, and semi-structured interviews with key informants including pharmacists, laboratory managers, hospital administrators, doctors, clinical officers, nurses, and research staff. The interviews lasted approximately 30 minutes and were conducted by pairs of assessment team members. No key informants refused participation. Salient concepts were recorded in real time by an assessment team member on semi-structured standardized forms. Although no formal thematic identification process was undertaken, paraphrased quotes and ideas were used to form and triangulate key findings. Observations of equipment, medicines, and facilities and abstractions of the clinical notes were also documented on standardized forms. All team members were trained on use of data report forms prior to conducting the assessment.

Hospitals were expected to have a full complement of essential equipment and medicines, and be able to provide a panel of basic laboratory tests as outlined below. No a priori cut-offs for acceptable adherence to guidelines or clinical staffing were set, as we are unaware of any validated norms that apply across the diversity of settings studied. However, results were compared to other published literature from high- and low-resource settings (Table B in S1 File).[12,18–27] The CHAIN Cohort study received ethical approval from Oxford University, University of Washington, and all Network sites. The funder had no role in the study or manuscript development.

### Hospital infrastructure & characteristics

Annual patient volumes, bed capacity, financing sources, participation in pre-service health worker education, blood bank, medical waste disposal and equipment sterilization facilities, and reliability of electricity and water supplies were assessed through key informant interviews and administrative reports. Radiology services were characterized by access to bedside or centralized ultrasound scanning and 24-hour x-rays, based on clinician interviews. Clinical laboratory staffing, external certification, availability of routine chemistries (electrolytes and creatinine), hematology (complete blood count), and microbiology (blood, cerebrospinal fluid, stool, urine, wound cultures) were catalogued during direct observation of laboratories and interviews with laboratory managers who were also asked about the frequency of reagent stockouts.

A high dependency score, consisting of nine characteristics assessed by direct observation, designed to capture high dependency or intensive care capacity (Table C in S1 File), was calculated at the facility level to reflect the highest capacity available to children at each institution. The high dependency score, and the essential equipment score (below), were also tabulated for all inpatient or pre-admission units which deliver substantial management, heuristically defined by capacity to provide injectable antibiotics, to children aged 2–23 months (i.e., CHAIN Cohort eligible).

### Essential medicines

The availability of potentially life-saving therapeutics, abstracted from the WHO Essential Medicines List and guideline-recommended nutritional products (ready-to-use therapeutic food (RUTF), ready-to-use supplementary food, milk formula-100 (F-100), and milk formula-75 (F-75))[28–30] was assessed through observation and key informant interviews with staff responsible for stock management. Informants were also asked if each item was subject to occasional or frequent stockouts. Therapeutics considered exchangeable within guidelines were condensed into a single-item category (e.g., normal saline and Ringer’s Lactate) and validated locally-made alternatives were accepted (e.g., milk suji formulations instead of F-75 and F-100 in Bangladesh).

### Essential equipment

An essential equipment inventory was adapted from published surveys and categorized into those used to identify acute illness and malnutrition, treat acute illness and malnutrition, and resuscitate children in cardiorespiratory decompensation.[12,13] Each item’s presence and functionality was assessed through observation and clinical staff interviews.

### Clinical care staffing

Pediatric care staff numbers were drawn from administrative data provided during interviews with senior physicians, charge nurses/matrons, or hospital administrators. Cadre per 1,000 pediatric admissions, and per 100 beds available to CHAIN participants (e.g., excluding neonatal units), were calculated in order to provide comparable figures across facilities of different sizes.

### Clinical management

Inpatient notes of recently discharged or deceased children aged 2–23 months admitted for acute illness were provided for our review by the sites. The CHAIN Cohort purposefully oversamples children with severe acute malnutrition (SAM). To ensure SAM management was adequately represented, therefore, approximately half of notes reviewed per hospital had a SAM diagnosis. Key indicators of guideline adherence were extracted from the first 48 hours of admission and grouped into seven core areas: 1) pulse oximetry and oxygen, 2) fluids, 3) anemia diagnosis and transfusion, 4) antibiotics, 5) malaria diagnosis and antimalarials, 6) nutritional assessment, SAM management, and young infant breastfeeding support, and 7) HIV testing.

Clinical practices were compared to WHO guidelines.[31,32] However, consistency with institutional or national recommendations was also considered guideline adherent (Table B in S1 File). Summary statistics, including estimating the proportion of children receiving adherent care, were calculated using Stata 14.2 (StataCorps, College Station, Tx). Finally, interviews with clinical staff were conducted to ascertain which discrepancies represented poor or colloquial documentation versus deviations from guideline recommended care.

## Results

### Hospital infrastructure and characteristics

Population and hospital characteristics: The CHAIN sites serve diverse populations across sub-Saharan Africa and South Asia with varied disease epidemiology (Table 1). Patient volumes also varied from 2,000 to 15,000 annual pediatric admissions per site, and hospitals had one to ten pediatric or neonatal wards. Seven hospitals had separate SAM units (Table 2).

**Table 1:** Population and disease epidemiology characteristics.

Hospital Name Region/Province Country	Site									
	Dhaka Dhaka Bangladesh	Matlab Chittagong Bangladesh	Civil Sindh Pakistan	Kilifi Coast Kenya	Mbagathi Nairobi Kenya	Migori Nyanza^a^ Kenya	Mulago Kampala Uganda	QECH Southern Malawi	Banfora Cascades Burkina Faso	Global
**Country indicators**										
GDP/capita (2016)	$1,359	$1,359	$1,444	$1,455	$1,455	$1,455	$580	$300	$627	$10,151
GDP/cap. rank (from lowest)	36^th^	36^th^	39^th^	40^th^	40^th^	40^th^	13^th^	2^nd^	14^th^	—
U5MR (2017)	38	38	81	49	49	49	55	64	89	43
MDG4 achieved	Yes	Yes	No	No	No	No	Yes	Yes	No	No
**Province indicators**										
Urban/Rural	Urban	Rural	Urban	Rural	Urban	Rural	Urban	Urban	Rural	—
Total fertility rate^b^	2.3	2.5	3.9	5.1	2.7	5.3	3.3	4.6	6.0	2.5
U5MR^b^	54	50	93	57	72	82	47	73	170	43
Malaria incidence^b^^,^^c^	0	0^d^	0.0–0.1	50–100	0.0–0.1	200–300	200–300	10–50	>300	94^f^
HIV prevalence, 15-49Y^b^	<0.1%	<0.1%	<0.1%	5%^e^	6%^e^	14%^e^	7%	15%	0.8%	1%
Wasting prevalence, <5Y^b^	12%	16%	14%	5%	3%	4%	4%	4%	12%	8%
Stunting prevalence, <5Y^b^	34%	38%	35%	31%	17%	26%	14%	37%	38%	24%

^a^Nyanza Province is no longer an administrative region, but demographic health and surveillance data are still aggregated at this level.

^b^Based on most recently available Demographic Health Survey data, therefore years vary.

^c^Incidence is per 1000 population. Highly malaria endemic is defined as >1 case per 1000 population.

^d^Malaria is endemic to eastern regions of Chittagong Division, but not Matlab.

^e^Kenyan HIV estimates listed are at the county rather than provincial level.

^f^Among at risk populations.

Abbreviations: Cap.: capita, GDP: gross domestic product, MDG4: Millennium Development Goal 4—Reduce the U5MR by two-thirds between 1990 and 2015, QECH: Queen Elizabeth Central Hospital, U5MR: under-five mortality rate (deaths per 1000 live births), Y: year-olds.

**Table 2:** Hospital characteristics.

	Hospitals								
	Dhaka Bangladesh	Matlab Bangladesh	Civil Pakistan	Kilifi Kenya	Mbagathi Kenya	Migori Kenya	Mulago Uganda	QECH Malawi	Banfora Burkina Faso
**Administration**									
Management	IRO	IRO	MOH	MOH	MOH	MOH	MOH	MOH	MOH
University hospital	—	—	Dow	—	—	—	Makerere	Malawi	Bobo-Dioulasso
Teaching hospital^a^	Yes	Yes	Yes	Yes	Yes	Yes	Yes	Yes	Yes
**Patient volumes**									
Ages considered pediatric	<5Y	<5Y	<13Y	<13Y	1M – 13Y	1M – 13Y	<18Y	<14Y	<15Y
Pediatric admissions/year	4,600	2,500	7,500	4,000	2,000	2,000	15,000	12,500	4,000
Pediatric & neonatal wards	4	3	5	2	1	1	5	10	4
Fees for pediatric care^b^	None	None	None	None	Daily fee + incurred costs	Daily fee + incurred costs	None	None	None
**Hospital Infrastructure**									
Medical waste disposal	Onsite incinerator	Onsite incinerator	Onsite incinerator	Onsite incinerator	Onsite incinerator	Onsite incinerator	Onsite incinerator	Onsite incinerator	Onsite incinerator
Equipment sterilization	Autoclave	Autoclave	Autoclave	Autoclave	Autoclave	Autoclave	Autoclave	Chlorination	Autoclave
Water supply reliability^c^	100%	100%	95%	90%	90%	50%	100%	90%	90%
Electricity reliability^c^	100%	100%	30%	100%	90%	95%	90%	90%	95%
X-ray radiography	24/7	Wk days 8–5	24/7	24/7	Wk days 8–5	24/7	24/7	24/7	24/7
Ultrasound scanner	In radiology	In radiology	In radiology	Bedside	No	In radiology	In radiology	Bedside	In radiology
Blood bank	No	No	On-site	On-site	Off-site	On-site	On-site	On-site	On-site
**Clinical Laboratory**									
Specimens per day	500	35	5000	120	250	300	500–650	500	150
In-hours staffing	60	6	150	1	5–8	10	26	14	3
Out-of-hours staffing	1	1	10	1	1	1	0	1	3
External certification	Yes	No	No	Yes	No	No	No	No	No
Routine testing services^d^	Yes	Yes	Yes	Yes	Yes^e^	Yes^e^	Yes	Yes	Yes
Bactec blood culture	Yes	No	Yes	Yes	No	No	Yes	No	No
**HDC score**	9/9	3/9	8/9	7/9	5/9	4/9	8/9	7/9	5/9

^a^Defined as including any clinician, nurse or nutritionist pre-service clinical clerkships.

^b^Incurred costs include for medications and diagnostics.

^c^Expressed as % of time reliably supplied according to key informants.

^d^Defined as capacity to perform basic biochemistries (urea, creatinine and standard electrolytes), complete blood count, and cultures on urine, cerebrospinal fluid, blood, wound, and stool specimens.

^e^Mbagathi and Migori had the full complement of clinical laboratory services assessed except the capacity to perform cultures on stool specimens.

Abbreviations: HDC: high dependency care, IRO: international research organization, M: month-olds, Wk: week, Y: year-olds.

Financing: All CHAIN sites receive funding from their respective health ministries, but five had additional sources. Matlab and Dhaka Hospitals receive support from bilateral donors. QECH Pediatric Department partners with a charity that provides funds to stock a pediatric pharmacy and supplement nursing staffing. At Civil Hospital, alumni and other local charities have financed numerous initiatives, including building and staffing a pediatric intensive care unit. The KEMRI-Wellcome Trust Research Programme supports Kilifi Hospital, including financing a high dependency unit, pediatric medicines, and salaries of researchers who also serve as clinical consultants.

Patient fees: Seven sites provide free care to children under five. Mbagathi and Migori Hospitals charge a subsidized daily inpatient fee of approximately $3.70 and $5.00, respectively, with added charges for investigations and medications. At all sites, families are asked to procure medicines and supplies from other sources during stockouts.

Clinical labs and radiology: All clinical laboratories have the infrastructure to provide basic services, however, reagent stockouts intermittently disrupted services at Mulago, Mbagathi, Migori, and Queen Elizabeth Central Hospitals. Sites unaffected by stockouts procured reagents outside of governmental systems, except for Civil Hospital. Clinical laboratories at two hospitals were externally certified. Ultrasonography was available at eight sites, while basic radiography was present at all sites, either on pediatric units or in radiology departments, with seven sites providing service on a 24-hour basis.

HDU/ICU: High dependency care scores range from 3–9 out of a maximum possible score of 9 (Table C in S1 File). All sites had a high dependency care area on a ward or a self-contained unit, devoted to the care of severe acute illness. All of these areas and units were at minimum close to a nursing station and with access to oxygen. QECH and Kilifi and Mulago Hospitals had dedicated high dependency units. Civil Hospital had a pediatric intensive care unit and Dhaka Hospital had a combined adult and pediatric intensive care unit managed by a pediatric intensivist.

### Essential medicines

Essential medicines were largely available during assessment visits, but frequent stockouts were reported (Table 3). F-75, F-100, and ReSoMal substitutes were prepared at Banfora, Mbagathi, and Kilifi Hospitals during stockouts, while the Migori Hospital nutrition team sought products from other counties.

**Table 3:** Essential medicines availability. Green—always in stock, yellow—occasional stockouts, red—frequent stockouts, black—never stocked.

Essential Medications	Dhaka Bangladesh	Matlab Bangladesh	Civil Pakistan	Kilifi Kenya	Mbagathi Kenya	Migori Kenya	Mulago Uganda	QECH Malawi	Banfora Burkina Faso
**Antibiotics**									
*Ampicillin/Benzyl penicillin*									
*Gentamicin*									
*Ceftriaxone*									
*Flucloxacillin*									
*Macrolide*									
*Ciprofloxacin*									
**1**^**st**^ **line antiretrovirals**									
**1**^**st**^ **line antituberculosis drugs**									
**Oral antimalarial**^**a**^									
**Injectable antimalarial**^**a**^									
**Emergency Medicines**									
*Adrenaline*									
*Atropine*									
*Intravenous or rectal short acting anti-convulsants*									
*Intravenous or oral phenobarbitol or phenytoin*									
**Intravenous fluids**									
*Isotonic fluids*									
*Dextrose 5% or 10%*									
*Dextrose (≥25%)*									
**Oral rehydration solution (ORS)**									
*Standard ORS*									
*ReSoMal*									
**Nutritional Therapeutics**									
*Milk formula 75 (F-75*^*b*^*)*									
*Milk formula 100 (F-100*^*b*^*)*									
*Ready-to-use therapeutic food*									
*Ready-to-use supplementary food*									

^a^Oral: artemisinin-based combination therapies, Injectable: artensunate, artemether, quinine.

^b^Milk suji equivalent used in Dhaka and Matlab hospitals

Some therapeutics were deliberately not stocked. Dhaka and Matlab Hospitals did not maintain anti-retrovirals because patients with suspected or confirmed HIV infection are referred to government facilities. Similarly, Banfora Hospital refers tuberculosis cases to a regional center and does not stock anti-tuberculosis medications. Milk suji formulations are used instead of F-75 and F-100 at Dhaka and Matlab Hospitals. Dhaka Hospital prepared nutrient dense therapeutic foods in lieu of RUTF, which has not yet been approved for use in Bangladesh, however, Matlab Hospital does not prepare these products. In Karachi, a high density diet is used in place of RUTF.

### Essential equipment

All hospitals had the range of essential equipment (Table 4) necessary for implementing guideline-based care at the time of their visit. Many sites had additional supplies (i.e., intubation kits, cardio-respiratory monitors, continuous positive airway pressure, and infusion pumps) allowing for enhanced care. Hospital composite scores were higher than individual wards’ scores (Table D in S1), due in large part to wards catering to specific conditions (e.g., nutritional rehabilitation units do not require chest drains) or equipment sharing between wards.

**Table 4:** Availability of equipment for assessment, treatment and resuscitation. Black = functioning equipment not available, Green = equipment available and functioning.

Equipment	Dhaka Bangladesh	Matlab Bangladesh	Civil Pakistan	Kilifi Kenya	Mbagathi Kenya	Migori Kenya	Mulago Uganda	QECH Malawi	Banfora Burkina Faso
**Pulse oximeter**									
**Cardio-respiratory monitor**									
**Sphygmomanometer**									
**Pediatric cuff**									
**Point of care glucose strips**									
**Torch/light source**									
**Otoscope**									
**Length board**									
**Scales basin**									
**Scales standing**									
**Mid-upper arm circumference tapes**									
**Suction equipment**									
**Oxygen**									
**Oxygen flow meter**									
**Nebulizer/spacer with face mask & inhaler**									
**Chest tubes**									
**Pediatric cannulas (24 or 22 gauge)**									
**Pediatric burette**									
**Intraosseous set**									
**Needles & syringes**									
**Intravenous giving sets**									
**Nasogastric tubes (gauges 8–10)**									
**Infusion pump**									
**Anti-epileptics on ward**									
**Antibiotics on ward**									
**Resuscitation table**									
**Airways**									
**Intubation set**									
**Infant-size bag-valve mask**									
**Child-size bag-valve mask**									
**Adrenaline**									
**Atropine**									

### Clinical care staffing

Pediatric human resources varied between hospitals (Table 5), but clinical and administrative staff at all sites reported overextended human resources to be the most prominent barrier to quality care. Doctors per 1,000 pediatric admissions ranged from 1.3–11.3, and were lower in African hospitals, where task shifting to clinical officers was common. Consultant-level physicians were more available at the urban sites, with the exception of Kilifi Hospital. QECH had fewer consultants per admission than the other urban centers, but key informants reported that senior doctor staffing levels are better than at other Malawian hospitals. Senior clinicians at all the African sites reported that the rotation of junior doctors and clinical officers exacerbate low staff levels by creating a constant need for training and reinforcement of best clinical practices.

**Table 5:** Pediatric care staffing at each hospital.

Cadre	Dhaka Bangladesh	Matlab Bangladesh	Civil Pakistan	Kilifi Kenya	Mbagathi Kenya	Migori Kenya	Mulago Uganda	QECH Malawi	Banfora Burkina Faso
	N (per 1,000 pediatric admissions)								
**Doctors**									
Consultants^a^	18 (3.9)	1 (0.4)	20 (2.7)	4 (1.0)	3 (1.5)	0	18 (1.3)	5 (0.4)	2 (0.5)
Pediatric post-graduate trainees	5 (1.1)	0	18 (2.4)	4 (1.0)	1 (1.5)	0	60 (4.0)	11 (0.9)	0
Medical officers	29 (6.3)	10 (4.0)	5 (0.7)	0	5 (2.5)	2 (0.7)	4 (0.3)	0	3 (0.8)
Interns	0	0	9 (1.1)	8 (2.0)	1 (0.5)	5 (1.7)	28 (1.8)	11 (0.9)	0
**Total**	**52 (11.3)**	**11 (4.4)**	**52 (6.9)**	**16 (4.0)**	**10 (6.0)**	**7 (2.4)**	**110 (7.3)**	**27 (2.2)**	**5 (1.3)**
**Mid-level Providers**^**b**^									
Fully-licensed	0	0	0	3 (0.8)	2 (1.0)	3 (1.0)	1 (0.1)	0	0
Interns	0	0	0	7 (1.8)	5 (2.5)	0	0	0	0
**Total**	**0**	**0**	**0**	**10 (1.8)**	**7 (3.5)**	**3 (1.0)**	**1 (0.1)**	**0**	**0**
**Nurses**	**88 (19.1)**	**17 (6.8)**	**42 (5.6)**	**32 (4.3)**	**12 (6.0)**	**7 (2.3)**	**85 (5.7)**	**34 (2.7)**	**44 (11.0)**
**Nursing assistants**	**8 (1.7)**	**0**	**9 (0.6)**	0	0	**0**	**9 (0.6)**	**15 (1.2)**	**0**
**Lay health workers**	**47 (10.2)**	**16 (6.4)**	**1 (0.1)**	**22 (5.5)**	**3 (1.5)**	**0**	**0**	**3 (0.2)**	**0**
**Nutritionists**^**c**^	**5 (1.1)**	**0**	**1 (0.1)**	**3 (0.8)**	**2 (1.0)**	**3 (0.0)**	**3 (0.2)**	**1 (0.1)**	**0**
	Per 100 beds^e^								
**Doctors**^**c**^	**78.8**	**11.2**	**30.1**	**27.6**	**21.7**	**26.9**	**36.5**	**15.5**	**3.5**
**Mid-level providers**^**b**^	**0**	**0**	**0**	**17.2**	**15.2**	**11.5**	**0.3**	**0**	**0**
**Nurses**^**d**^	**133.3**	**17.3**	**24.3**	**55.2**	**26.1**	**26.9**	**27.6**	**19.5**	**12.3**
**Nursing assistants**	**12.1**	**0**	**5.2**	**0**	**0**	**0**	**2.9**	**8.6**	**0**
**Lay health workers**	**71.2**	**16.3**	**0.6**	**37.9**	**6.5**	**0**	**0**	**1.7**	**0**
**Nutritionists**^**c**^	**7.6**	**0**	**0.6**	**5.2**	**4.3**	**11.5**	**1.0**	**0.6**	**0**

^a^Clinical full time equivalent (i.e., time allocated to research not included). Remaining cadres are full time clinical staff.

^b^Clinical officers in Africa.

^c^ Typical beds-per-doctor on service during day-time hours were: Dhaka 4.3, Matlab 8.0, Civil 5.7, Kilifi 9.7, Mbagathi 11.5, Migori 6.5, Mulago 9.6, QECH 7.3, Banfora 22.0, with night-time ratios of: Dhaka 39.0, Matlab 28.0, Civil 14.0, Kilifi 58.0, Mbagathi 46.0, Migori 26.0, Mulago 44.0, QECH 58.0, Banfora 67.0.

^d^Typical beds-per nurse during day-time hours were: Dhaka 9.8, Matlab 7.0, Civil 5.7, Kilifi 9.7, Mbagathi 23.0, Migori 26.0, Mulago 16.2, QECH 5.8, Banfora 16.8, with night-time ratios of: Dhaka 13.0, Matlab 14.0, Civil 6.7, Kilifi 9.7, Mbagathi 23.0, Migori 26.0, Mulago 34.2, QECH 19.3, Banfora 16.8.

^e^Available to CHAIN patients (e.g., not including neonatal units).

Nurse staffing levels were between 1.8–19.1 per 1,000 pediatric admissions. Key informants at all sites reported that inadequate nurse staffing levels limit care such as anthropometric screening, nutritional counselling and breast-feeding support. Nutritionists or dieticians were employed at all sites excluding Banfora and Matlab Hospitals, and nursing assistants and lay health workers were variably utilized.

All sites participated in health worker undergraduate or diploma training programs, and the African sites’ senior clinicians and nurses noted that students are an important supplement to human resource capacity, albeit inconsistent due to fluctuating trainee schedules.

### Clinical management

Institutional or national adaptations of WHO guidelines were available as wall charts or in handbooks on the ward at each site. The clinical notes of 261 children were reviewed (22–38 per site). Multiple diagnoses were noted in 115 cases. Cumulatively, there were seven shock, 17 sepsis, 47 malaria, 70 pneumonia, 106 diarrhea, and 119 SAM diagnoses. The median age at admission was nine months (interquartile range (IQR): 6–15). Twenty-nine (11%) children died during hospitalization, the median time from admission to death was three days (IQR 2–8). Excluding inpatient deaths, median length of stay was four days (IQR: 2–7).

#### Pulse oximetry and oxygen

Pulse oximetry was documented for 53/70 (76%) of children with pneumonia diagnosed at admission. Hypoxia (oxygen saturation <90% in room air)[29] was noted for 8/53 (12%) children with pneumonia diagnoses Oxygen orders were noted for three-quarters of these children (6/8). Another 11 children with pneumonia diagnoses were noted to be on oxygen, but had saturations above 90% or no recorded oxygen saturations, during their admission examination.

#### Fluids

Intravenous fluid boluses are indicated for intravascular volume expansion in children with severe dehydration or shock using isotonic fluids at 20 ml/kg (repeated as needed), or for managing hypoglycemia with 10% dextrose at 5 ml/kg.[29] Twenty-seven children (10%) had documented prescriptions of fluid boluses. Blood glucose levels were rarely recorded, though among those noted as having received boluses, 17/27 (63%) appeared to be for the prevention or management of hypoglycemia, suggested by the use of 10% dextrose water solution at a median volume of 4.5 ml/kg (IQR: 3.0–4.9). The remaining 10 bolus volumes were consistent with volume expansion (median volume of 34.4 ml/kg (IQR: 30–60)). Nine of these 10 boluses were isotonic fluids, the other was dextrose saline. Dehydration status was poorly documented, but 9/10 (90%) of the children who received volume expansion boluses had documented potential indications (9 diarrhea, 3 shock, 1 sepsis).

Intravenous or oral rehydration was noted for 96/106 (91%) children with diarrhea, and oral rehydration solution (ORS) was prescribed for 86/106 (81%). All 23 children with diarrhea and SAM co-diagnoses were correctly prescribed ReSoMal or standard ORS according to local guidelines.

#### Anemia diagnosis and transfusion

Hemoglobin concentrations were recorded in the notes of 167 (64%) children and in 96/148 (65%) of those admitted to sites with high malaria endemicity (African sites excluding Mbagathi Hospital in Nairobi). The WHO anemia diagnostic criteria (hemoglobin <11g/dl)[29,31] was met by 122/167 children, but only 23 (19%) had an explicit diagnosis in their clinical notes. Among children with severe anemia (hemoglobin <7g/dl), 10/28 (36%) had a corresponding diagnosis.

The WHO recommends blood transfusion for hemoglobin <4.0 g/dl, hemoglobin ≥4.0 and <6.0 g/dl with clinical signs of shock, dehydration, respiratory acidosis, impaired consciousness, heart failure, or severe hyperparasitemia. Persistent shock unresponsive to appropriate intravenous fluid resuscitation is also indication for transfusion regardless of hemoglobin status.[29,31] For the purposes of this assessment, transfusions were considered indicated if there was documentation of shock or of a hemoglobin concentration <6g/dl. Thirty-five children had transfusions ordered, 31 (89%) of whom had a documented hemoglobin result, and 23/35 (66%) had a documented potential indication including 18/35 (51%) with hemoglobin <6 g/dl and an additional five with shock diagnoses. Clinicians at three sites reported challenges obtaining blood products (Kilifi, Dhaka, Matlab Hospitals), however, informants at all but Kilifi Hospital indicated that this rarely affected care.

#### Antibiotic management

Antibiotics were prescribed to 168/170 (99%) children with a documented indication at admission. Overall, 128/170 (75%) of these regimens were adherent to guidelines. Adherent regimens were prescribed to 78/106 (74%) with SAM, 60/70 (86%) with pneumonia, 16/17 (94%) with sepsis, and 8/12 (67%) with meningitis.

Antibiotics are not recommended for malaria or diarrhea without dysentery or suspected cholera unless there is a comorbidity necessitating antibiotics. Among 108 children with a diagnosis of diarrhea, 105 (97%) were prescribed antibiotics. However, 67 had SAM, 17 had another comorbidity warranting antimicrobials, and one had dysentery. Twenty (90%) of the 23 children with diarrhea and no documented indication for antibiotics were prescribed antibiotics.

Thirty-nine (83%) of 47 children with malaria diagnoses were prescribed antibiotics, including 20/27 (74%) without documented comorbid indications for antibiotics. Thirteen of these 20 (65%) had a recorded positive malaria test, one had a negative malaria test noted, and six did not have a documented malaria test.

#### Malaria diagnosis and antimalarials

At highly malaria endemic sites, 49/68 (72%) children with febrile temperature measurements had malaria test results recorded, predominantly by parasite smears. Thirty-three children without documented fever also had recorded malarial test results. Thirty-seven of the 82 (45%) test results were noted to be positive, and all but one child with confirmed malaria were prescribed antimalarials. One child with a negative result and 10 without documented test results were prescribed an antimalarial. All antimalarials prescribed were guideline-adherent.

#### Nutritional assessment, SAM management, and breastfeeding support for young infants

Six sites screened all pediatric inpatients for SAM, while three did so based on clinical suspicion only. Assessment was by Gomez classification at one site, weight-for-height Z score the remainder, and six sites additionally employed mid-upper arm circumference (MUAC). Anthropometric status was noted in 136 (52%) patient notes, though at two sites with systematic screening and electronic capture systems, the values were not obviously visible, and were therefore missed by the assessment team. The WHO recommends F-75 at refeeding rates of 130 mls/kg/day for SAM (100mls/kg/day for kwashiorkor) acute phase management.[29] Ninety-three (78%) of the 119 patients diagnosed with SAM had recorded feeding rates. The median initial feeding rate was 128 ml/kg/day (IQR: 107–133).

For young infants (age <6 months) with SAM, eight hospitals used dilute F-100 to supplement breastmilk, and one never supplemented breastmilk (QECH). All sites offered breastfeeding support, irrespective of nutritional status. Six hospitals provided relactation support for mothers of young infants who had ceased breastfeeding. Five hospitals did not have materials or infrastructure (e.g., play room or toys) to provide play therapy recommended for psychosocial stimulation for inpatient children with SAM.[30,33]

The management of moderate acute malnutrition (MAM) varied across sites. Migori Hospital managed these children according to SAM refeeding recommendations. At Dhaka and Matlab Hospitals, all pediatric inpatients received specific milk-based or lactose-free feeds, including those with MAM. QECH and Kilifi, Mbagathi, and Mulago Hospitals provided a two-week supply of corn-soy blend at discharge, while in Banfora Hospital caregivers received nutritional education without food supplementation. No specific MAM interventions were delivered at Civil Hospital.

#### HIV testing

The African sites aimed for universal HIV screening of pediatric admissions, but only 65/173 (38%) children admitted to these hospitals had HIV test results documented in their notes. Interviewees attributed this deficit to concealment of results (e.g., locally specific codes or log-books outside of patient notes). Three sites reported interruptions in universal screening and follow-up care due to test kit stockouts, inadequate staffing, or siloed HIV programs. The Asian sites tested or referred for testing based on clinical suspicion only, and no test results were identified in our notes review.

## Discussion

All CHAIN Network sites had the infrastructure, equipment, and access to guidelines necessary to provide care consistent with current recommendations. Several sites had enhanced care capacity, such as a high dependency unit. Given that all sites’ facilities and equipment enable their ability to meet minimum care standards as defined by WHO pediatric inpatient care guidelines, capacity above this expectation is a welcome addition to the Network. The additional resources were largely supported by charitable donations, philanthropic support, or research infrastructure that play a critical role at five sites. These hospitals were also least affected by stockouts of laboratory reagents and medicines, as these commodities could be supplemented by research or charitable partnerships (QECH and Civil and Kilifi Hospitals) or they relied exclusively on non-governmental procurement channels (Dhaka and Matlab Hospitals). The other four sites reported laboratory reagents and medicines to be common and a barrier to quality patient care.

The clinical notes review demonstrated that adherence to recommended care was inconsistent. Perfect guideline adherence may be undesirable, as some deviations reflect appropriate tailoring of care to individual patients. The observed frequency of guideline adherence prior to initiating the CHAIN Cohort study was comparable or better than other studies in high- and low-income settings.[12,18–23,27] However, several areas for improvement were identified. Three areas of suboptimal adherence, or poor record keeping, were anemia diagnosis, HIV and malaria testing, and hypoxemia assessment and treatment. Medical note reviews cannot discriminate inadequate record keeping from poor practice. However, this exercise does highlight the importance of adequate record-keeping, which can be enhanced by pre-formatted structured medical notes, and can lead to improvements in clinical care.[18,34,35]

Antibiotics were almost universally prescribed when indicated and adherence to recommended regimens was comparable or higher than other studies.[12,20–27] Similar to other published data, antibiotics were also frequently prescribed to children without an indication.[24–26] A high proportion of antibiotic prescriptions for children with diarrhea and malaria could be explained by differential diagnoses requiring empiric antibiotic treatment. This finding echoes a recent large observational study in Kenya that found 84% of children with diarrhea and dehydration were diagnosed with at least one additional condition.[17] This suggests that helping clinicians confidently exclude these differential diagnoses could considerably improve antibiotic stewardship. The CHAIN Network now provides malaria and HIV screening to all enrolled children, chest radiographs to children when indicated, and microbiological diagnostic support which may increase the specificity of clinical diagnoses. However, a recent Cochrane review demonstrated that introduction of diagnostic tests, such as malaria rapid diagnostics, can have unpredictable influences on antimicrobial utilization patterns.[26]

Malnutrition screening protocols varied across sites, with several hospitals neither using MUAC nor screening all pediatric admissions. Some key informants expressed concern that expanding indications for specialist nutrition services would increase strain on already overburdened clinical staff. Concerns were raised by some interviewees about introducing MUAC, including suitability for inpatient settings, especially at national or regional referral hospitals. Children with SAM and low MUAC are typically a higher risk group than those identified by WHZ alone;[36–38] the CHAIN Network will work with all sites to implement routine screening using both anthropometrics.

Site leaders related many aspects of inconsistent guideline adherence to low doctor- and nurse-to-patient ratios, frequent rotation of trainees, and difficulty providing adequate supervision of junior clinicians. Insufficient nursing personnel was universally noted by site leadership to be problematic, this was consistent even across a wide range of nurse-to-patient ratios. The WHO offers advice on numbers of skilled health personnel needed at the population level,[39] but we are unaware of staffing recommendations at the facility level. Previous studies, typically in high-income countries, have used staff-per-bed ratios to represent human resource capacity, assuming that hospital beds are proportional to patient volumes.[40–42] Facilities in resource-limited settings often experience patient volumes beyond their bed capacity, and consequently double or triple patients-per-bed, add beds outside designated ward areas, or use floor space to accommodate more patients. In these facilities, staff-per-1000 admissions may be a better metric for patient volume. The Access, Bottlenecks, Costs and Equity (ABCE) project, found that 625 hospitals in Kenya, Uganda, and Zambia had a mean of 7.2, 6.1, and 3.8 doctors-per-100 beds, respectively. They also identified a mean of 49.3, 46.1, and 22.0 nurse-per-100 beds. The CHAIN sites had similar doctor-, but lower nurse-to-bed ratios, which may reflect their public ownership, whereas ABCE included private hospitals.[43] Our assessment, and the ABCE analysis, noted substantial variation in human resources between sites. Quality assessment and improvement initiatives would benefit from institutional-level staffing benchmarks that represent efficiency and quality of care.

Our evaluation had several limitations. First, sites were included because they are CHAIN Network members and were not selected as a representative sample of low- and middle-income country hospitals. The notes review was a convenience sample and was not powered to make definitive statements about clinical practices at each site, test association between facility level factors and guideline adherence, or assess the impact of non-adherence on mortality. Additionally, a clinical notes review does not capture many important aspects of care, such as the quality of health education provided to caretakers, and cannot differentiate weak documentation from poor practice. Next, we assessed inappropriate use of intravenous fluid boluses, blood transfusions, and antibiotics in the context of diarrhea, because these treatments may cause adverse effects, especially if used inappropriately. However, we did not assess the role of non-adherent “overuse” of diagnostics or therapeutics in consuming, and thereby diverting, limited resources from utilization for appropriate care. Finally, assessment of stockouts may have been subject to recall bias, social desirability bias may have influenced aspects of the qualitative data, and inter-rater reliability of clinical notes abstraction were not assessed. Despite these limitations, this study utilized a multiple assessment methodology which builds on previous quality assessment work and provides a template for a systematic assessment of pediatric inpatient capacity and care across diverse settings in multiple countries.

## Conclusion

The CHAIN Network sites had the infrastructure and equipment necessary to implement a standard of care that met or exceeded WHO recommendations. Despite this capacity, adherence to standardized care guidelines among children treated prior to CHAIN research activities was inconsistent. Some discrepancies might reflect appropriate tailoring of care to individual patients. Stretched human resources and stockouts of medicines, nutritional therapeutics, and laboratory supplies were the most commonly reported barriers to guideline implementation. Clinical staffing benchmarks at the facility-level would greatly enhance quality assessment and improvement efforts. Finally, health worker time requirements should be assessed when testing implementation of novel inpatient diagnostics and treatments, as it may be a critical determinant of effectiveness outside of clinical trial conditions.

## Supporting information

S1 File Details of data collection, comparable adherence estimates, high dependency scores and ward specific equipment scores. (DOCX)

S1 Appendix List of CHAIN network authors. (DOCX)
