# Supplementary material for: A mixed method multi-country assessment of barriers to implementing pediatric inpatient care guidelines
Source: PLoS One. 2019 Mar 25;14(3):e0212395. doi: 10.1371/journal.pone.0212395 (PMC6433255; doi:10.1371/journal.pone.0212395)
Supplement: S1 File — (DOCX) [file pone.0212395.s001.docx]

Table A. Variables collected and methods of assessment.

| **Characteristic** | **Method of Assessment** |
| --- | --- |
| GENERAL HOSPITAL INFORMATION | |
| Catchment area population characteristics | Literature searches |
| What is the population size in the hospital's catchment area? | Key informant interviews; hospital reports |
| What level hospital is this? | Key informant interviews; hospital reports |
| How many beds are there in the hospital? | Key informant interviews |
| How many pediatric beds are available in the hospital? | Key informant interviews |
| How many pediatric admissions are there per year in this hospital? | Key informant interviews |
| What is the overall mortality rate of pediatric patients in this hospital? (percentage) | Key informant interviews |
| What is the % of children who die within the 1st 24 hours? | Key informant interviews |
| What is the average length of stay of pediatric patients? | Key informant interviews |
| What is the maximum and minimum age of pediatric patients in the wards? | Key informant interviews |
| Patient flow | Key informant interviews |
| Admission procedures | Key informant interviews |
| Ward transfer procedures | Key informant interviews |
| Discharge decision making | Key informant interviews |
| Follow-up procedures | Key informant interviews |
| Where do children get admitted from? | Direct observation; key informant interviews |
| Who decides whether a child is admitted? | Direct observation; key informant interviews |
| Who decides whether a child is admitted to ICU or HDU if any? | Direct observation; key informant interviews |
| Who decides id a child is admitted to malnutrition ward if any? | Direct observation; key informant interviews |
| Who decides if a child's admission is to step up to ICU/HDU from a general ward? | Direct observation; key informant interviews |
| Who decides if a child's admission is to step down to general ward from ICU/HDU? | Direct observation; key informant interviews |
| Who decides when a child is to be discharged? | Direct observation; key informant interviews |
| Where do children with SAM get followed up after discharge? | Direct observation; key informant interviews |
| Who weighs and measures length and/or MUAC on admission? | Direct observation; key informant interviews |
| Where do children with HIV infection get followed up after discharge? | Direct observation; key informant interviews |
| Who decides on transfer of children to malnutrition ward from different wards? | Direct observation; key informant interviews |
| What kinds of fees are incurred for admission of children under 5? List and include amounts. | Key informant interviews |
| Does this hospital have access to electronic medical records (EMR)? | Key informant interviews |
| Is internet available on the pediatric ward? | Key informant interviews |
| If yes, how regularly? | Key informant interviews |
| Do pediatric patients have any problems accessing radiology (e.g. CXR)? | Key informant interviews |
| Is bedside ultrasound available on the pediatric wards? | Key informant interviews |
| If yes, what is it used for? | Key informant interviews |
| Does the pediatric ward have access to running water supply? | Key informant interviews |
| If yes, describe the source: | Key informant interviews |
| How regularly is the water running/available? | Key informant interviews |
| Does the pediatric ward have constant access to electricity? | Key informant interviews |
| If no, how often does the electricity not run? | Key informant interviews |
| What type of sterilization equipment is used to sterilize instruments and/or supplies used on the pediatric ward? | Key informant interviews |
| What type of waste disposal facility is used for medical waste from the pediatric ward? | Key informant interviews |
| Is oxygen available on the pediatric ward? | Key informant interviews |
| If yes, how often is the supply disrupted? | Key informant interviews |
| If yes, is the oxygen piped? | Key informant interviews |
| Does this hospital have blood bank facilities? | Key informant interviews |
| Are mechanical ventilators available for pediatric patients? | Key informant interviews |
| Other hospital infrastructure | Key informant interviews |
| Number of Midlevel/Clinical Officers? | Key informant interviews |
| Number of Midlevel/Clinical Officers in the day time? | Key informant interviews |
| Number of Midlevel/Clinical Officers at night? | Key informant interviews |
| Number of Senior Midlevel/Senior Clinical Officers? | Key informant interviews |
| Number of Senior Midlevel/Senior Clinical Officers in the day time? | Key informant interviews |
| Number of Senior Midlevel/Senior Clinical Officers at night? | Key informant interviews |
| Number of full time House Officers/Interns? | Key informant interviews |
| Number of full time House Officers/Interns during the day? | Key informant interviews |
| Number of full time House Officers/Interns during the night? | Key informant interviews |
| Number of full time Medical Officers? | Key informant interviews |
| Number of Medical Officers during the day? | Key informant interviews |
| Number of Medical Officers during the night? | Key informant interviews |
| Number of full time Senior Medical Officers/Residents/Postgrads? | Key informant interviews |
| Number of Senior Medical Officers/Residents/Postgrads during the day? | Key informant interviews |
| Number of Senior Medical Officers/Residents/Postgrads at night? | Key informant interviews |
| Number of full time consultants? | Key informant interviews |
| Number of consultants during the day? | Key informant interviews |
| Number of night time consultants? | Key informant interviews |
| Number of registered nurses? | Key informant interviews |
| Number of registered nurses during the day? | Key informant interviews |
| Number of registered nurses at night? | Key informant interviews |
| Number of full time nurse assistants? | Key informant interviews |
| Number of nurse assistants during the day? | Key informant interviews |
| Number of nurse assistants at night? | Key informant interviews |
| Number of full time volunteer nurses? | Key informant interviews |
| Number of volunteer nurses during the day? | Key informant interviews |
| Number of volunteer nurses at night? | Key informant interviews |
| Number of full time lay health workers? | Key informant interviews |
| Number of lay health workers during the day? | Key informant interviews |
| Number of lay health workers at night? | Key informant interviews |
| Number of full time nutritionists? | Key informant interviews |
| Number of nutritionists during the day? | Key informant interviews |
| Number of nutritionists at night? | Key informant interviews |
| Number of occupational or physical therapists? | Key informant interviews |
| Number of play therapists? | Key informant interviews |
| Number of social workers? | Key informant interviews |
| Number of cleaners? | Key informant interviews |
| Number of kitchen staff? | Key informant interviews |
| Other staff? Note cadre and number of people. | Key informant interviews |
| EQUIPMENT AND SUPPLIES ON THE WARDS (verify that all equipment is in working order) | |
| Resuscitation table | Direct observation; key informant interviews |
| Infant-size bag valve mask | Direct observation; key informant interviews |
| Child-size bag valve mask | Direct observation; key informant interviews |
| Weighing scales, basin | Direct observation; key informant interviews |
| Weighing scales, standing | Direct observation; key informant interviews |
| MUAC tapes | Direct observation; key informant interviews |
| Length board | Direct observation; key informant interviews |
| Nebulizer | Direct observation; key informant interviews |
| Spacer and infant face mask for use with inhaler | Direct observation; key informant interviews |
| Spacer and child face mask for use with inhaler | Direct observation; key informant interviews |
| Oxygen | Direct observation; key informant interviews |
| Oxygen flow meter | Direct observation; key informant interviews |
| Pulse oximeter | Direct observation; key informant interviews |
| Sphygmomanometer | Direct observation; key informant interviews |
| Pediatric blood pressure cuff | Direct observation; key informant interviews |
| Torch | Direct observation; key informant interviews |
| Otoscope | Direct observation; key informant interviews |
| Defibrillator | Direct observation; key informant interviews |
| Nasogastric tubes (gauges 8-10) | Direct observation; key informant interviews |
| Pediatric burettes | Direct observation; key informant interviews |
| Infusion pump | Direct observation; key informant interviews |
| IV giving sets | Direct observation; key informant interviews |
| Pediatric cannulas (22 or 24 gauges) | Direct observation; key informant interviews |
| Needles and syringes | Direct observation; key informant interviews |
| Suction equipment | Direct observation; key informant interviews |
| Cardio-respiratory monitor | Direct observation; key informant interviews |
| Intubation sets | Direct observation; key informant interviews |
| Intraosseous sets | Direct observation; key informant interviews |
| Scalp vein sets | Direct observation; key informant interviews |
| Chest tubes | Direct observation; key informant interviews |
| Point of care glucose strips | Direct observation; key informant interviews |
| Epinephrine | Direct observation; key informant interviews |
| Atropine | Direct observation; key informant interviews |
| Playroom | Direct observation; key informant interviews |
| Toys | Direct observation; key informant interviews |
| Other basic equipment | Direct observation; key informant interviews |
| THERAPEUTICS AVAILABLE IN HOSPITAL (E.G., PHARMACY) | |
| >= 25% Dextrose (intravenous) | Direct observation; key informant interviews |
| 10% Dextrose | Direct observation; key informant interviews |
| 5% Dextrose | Direct observation; key informant interviews |
| Normal Saline | Direct observation; key informant interviews |
| Ringer’s Lactate | Direct observation; key informant interviews |
| Half-strength Darrow’s | Direct observation; key informant interviews |
| ORS, Standard | Direct observation; key informant interviews |
| ReSoMal | Direct observation; key informant interviews |
| Potassium Chloride | Direct observation; key informant interviews |
| Sodium Hydrogen Carbonate | Direct observation; key informant interviews |
| Water for injection | Direct observation; key informant interviews |
| Whole blood | Direct observation; key informant interviews |
| Platelets | Direct observation; key informant interviews |
| Zinc tabs | Direct observation; key informant interviews |
| Multiple micronutrient packets | Direct observation; key informant interviews |
| Vitamin A | Direct observation; key informant interviews |
| Iron tabs/syrup | Direct observation; key informant interviews |
| F-100 | Direct observation; key informant interviews |
| F-75 | Direct observation; key informant interviews |
| Term Formula | Direct observation; key informant interviews |
| Benzyl penicillin | Direct observation; key informant interviews |
| Amoxicillin syrup | Direct observation; key informant interviews |
| Gentamicin | Direct observation; key informant interviews |
| (Flu)cloxacillin | Direct observation; key informant interviews |
| Ceftriaxone | Direct observation; key informant interviews |
| Chloramphenicol | Direct observation; key informant interviews |
| Ciprofloxacin | Direct observation; key informant interviews |
| Amoxicillin - clavulanate | Direct observation; key informant interviews |
| Ampicillin | Direct observation; key informant interviews |
| Azithromycin | Direct observation; key informant interviews |
| Doxycycline | Direct observation; key informant interviews |
| Erythromycin | Direct observation; key informant interviews |
| Metronidazole | Direct observation; key informant interviews |
| Nitrofurantoin | Direct observation; key informant interviews |
| Sulfa-trimethoprim/Cotrimaxazole | Direct observation; key informant interviews |
| Cefalexin | Direct observation; key informant interviews |
| Nystatin | Direct observation; key informant interviews |
| Aciclovir | Direct observation; key informant interviews |
| Abacavir (ABC) | Direct observation; key informant interviews |
| Lamivudine (3TC) | Direct observation; key informant interviews |
| Stavudine (d4T) | Direct observation; key informant interviews |
| Zidovudine (ZDV or AZT) | Direct observation; key informant interviews |
| Efavirenz | Direct observation; key informant interviews |
| Tenofovir (TDF) | Direct observation; key informant interviews |
| Emtricitabine (FTC) | Direct observation; key informant interviews |
| Lopinavir (LPV/r) | Direct observation; key informant interviews |
| Nevirapine (NVP) | Direct observation; key informant interviews |
| Atazanavir | Direct observation; key informant interviews |
| Artemether + lumefantrine | Direct observation; key informant interviews |
| Artesunate + amodiaquine | Direct observation; key informant interviews |
| Artesunate +mefloquine | Direct observation; key informant interviews |
| Chloroquine | Direct observation; key informant interviews |
| Quinine | Direct observation; key informant interviews |
| Sulfadoxine + pyrithemamine (Fansidar) | Direct observation; key informant interviews |
| Diazepam | Direct observation; key informant interviews |
| Phenytoin | Direct observation; key informant interviews |
| Phenobarbitone | Direct observation; key informant interviews |
| Adrenaline | Direct observation; key informant interviews |
| Salbutamol | Direct observation; key informant interviews |
| Hydrocortisone | Direct observation; key informant interviews |
| Dexamethasone | Direct observation; key informant interviews |
| Prednisolone/ Prednisone | Direct observation; key informant interviews |
| Digoxin | Direct observation; key informant interviews |
| Lasix | Direct observation; key informant interviews |
| Mannitol | Direct observation; key informant interviews |
| Isoniazid | Direct observation; key informant interviews |
| Rifampicin | Direct observation; key informant interviews |
| Pyrazinamide | Direct observation; key informant interviews |
| Ethambutol | Direct observation; key informant interviews |
| List first line HIV drugs/ antiretrovirals used for pediatric patients at this facility | Direct observation; key informant interviews |
| List first line TB drugs used for pediatric patients at this facility | Direct observation; key informant interviews |
| LABORATORY FACILITIES | |
| What types of labs are you able to obtain for clinical purposes? | Key informant interviews; direct observation |
| What is the typical number of lab specimens processed/day? | Key informant interviews; direct observation |
| What are the machines used for each lab? | Key informant interviews; direct observation |
| For microbiology, what types of specimens can you culture? | Key informant interviews; direct observation |
| For blood culture, do you use Bactec? | Key informant interviews; direct observation |
| For microbiology, do you use any type of external quality validation? | Key informant interviews; direct observation |
| How is malaria tested for? | Key informant interviews; direct observation |
| Who certifies the lab? | Key informant interviews; direct observation |
| How frequently is lab certification done? | Key informant interviews; direct observation |
| What is your lab capacity at night / on weekends? | Key informant interviews; direct observation |
| Any other lab observed? | Key informant interviews; direct observation |
| Name of additional lab and notes on it | Key informant interviews; direct observation |

Table A. Part 2: Clinical notes review.

| **Variable** | **Method of Assessment** |
| --- | --- |
| Age in months | Clinical note review (data extraction) |
| Date of admission | Clinical note review (data extraction) |
| Admission weight (in kg) | Clinical note review (data extraction) |
| Screened with MUAC | Clinical note review (data extraction) |
| MUAC reading | Clinical note review (data extraction) |
| Screened with WHZ | Clinical note review (data extraction) |
| WHZ reading | Clinical note review (data extraction) |
| Admitting diagnosis | Clinical note review (data extraction) |
| High dependency or intensive care unit management | Clinical note review (data extraction) |
| Discharge disposition | Clinical note review (data extraction) |
| Discharge diagnosis | Clinical note review (data extraction) |
| Length of stay (# nights spend in hospital) | Clinical note review (data extraction) |
| Referral | Clinical note review (data extraction) |
| Fever on admission | Clinical note review (data extraction) |
| Malaria testing |  |
| If yes, test results | Clinical note review (data extraction) |
| If yes, type of malaria test | Clinical note review (data extraction) |
| Severe acute malnutrition (SAM) diagnosis | Clinical note review (data extraction) |
| Moderate acute malnutrition (MAM) diagnosis | Clinical note review (data extraction) |
| Diarrhea diagnosis | Clinical note review (data extraction) |
| Pneumonia diagnosis | Clinical note review (data extraction) |
| Severe pneumonia diagnosis | Clinical note review (data extraction) |
| Malaria diagnosis | Clinical note review (data extraction) |
| Anemia diagnosis | Clinical note review (data extraction) |
| Severe dehydration diagnosis | Clinical note review (data extraction) |
| Shock diagnosis | Clinical note review (data extraction) |
| Hemoglobin testing on admission | Clinical note review (data extraction) |
| If yes, note value | Clinical note review (data extraction) |
| Known HIV positive status? | Clinical note review (data extraction) |
| HIV testing performed | Clinical note review (data extraction) |
| If yes, note HIV test result | Clinical note review (data extraction) |
| Intravenous fluids (IVF) | Clinical note review (data extraction) |
| If yes, note types of IVFs given in the first 24 hours: Include volume administered for each one over specific time frame (e.g. NS 200ml/10 minutes) | Clinical note review (data extraction) |
| Oral rehydration solution (ORS) | Clinical note review (data extraction) |
| If yes, note types of ORS given in the first 24 hours: Include volume administered for each one over specific time frame (e.g. standard ORS 100ml/4 hours) | Clinical note review (data extraction) |
| Milk formula 75/100 | Clinical note review (data extraction) |
| If yes, milk formula feeding rate in the first 24 hours of admission in ml/hour | Clinical note review (data extraction) |
| Feeds recorded according to actual volume consumed (i.e. leftovers charted) | Clinical note review (data extraction) |
| Feeds recorded according to actual time provided | Clinical note review (data extraction) |
| Antibiotics in the 1st 48 hours of admission | Clinical note review (data extraction) |
| If yes, list antibiotics | Clinical note review (data extraction) |
| Antimalarials in the 1st 48 hours of admission | Clinical note review (data extraction) |
| If yes, list antimalarials | Clinical note review (data extraction) |
| Respiration rate recorded | Clinical note review (data extraction) |
| If yes, note first value recorded | Clinical note review (data extraction) |
| If yes, note frequency of observations of respiration rates | Clinical note review (data extraction) |
| Heart rate recorded | Clinical note review (data extraction) |
| If yes, note first value recorded | Clinical note review (data extraction) |
| If yes, note frequency of observations of heart rates | Clinical note review (data extraction) |
| Blood pressure recorded | Clinical note review (data extraction) |
| If yes, note first value recorded | Clinical note review (data extraction) |
| If yes, note frequency of observations of blood pressures | Clinical note review (data extraction) |
| Pulse oximetry recorded | Clinical note review (data extraction) |
| If yes, note first value recorded | Clinical note review (data extraction) |
| If yes, note frequency of observations of oximetry | Clinical note review (data extraction) |
| Oxygen administered | Clinical note review (data extraction) |
| If yes, note number of hours from admission to when O2 administration noted | Clinical note review (data extraction) |
| Transfusion administered | Clinical note review (data extraction) |
| If yes, list all products | Clinical note review (data extraction) |
| If yes, record volume and duration of each product (e.g., 250 ml of whole blood over 1 hour) | Clinical note review (data extraction) |
| Additional notes | Clinical note review (data extraction) |

Abbreviations: CPAP – Continuous positive airway pressure, A&E – Accident and Emergency, ED – Emergency department, HDU – High dependency unit, Hosp. – Hospital, ICU – Intensive care unit, IVF – Intravenous fluids, LSW – Long stay ward, NRU – Nutrition Unit, MCH – Maternal Child Health, PICU – Pediatric intensive care unit, QECH – Queen Elizabeth Central Hospital

Table B. Adherence definitions and results.

| **Area** | **Definition of adherence**^a^ | **Observed estimates** | **Previously published estimates** |
| --- | --- | --- | --- |
| Oxygen | Pulse oximetry for pneumonia  Oxygen for hypoxia (oxygen saturations < 90%) | 76%  75%^b^ | --  35% & 37%^18^ |
| Fluids | ORS for diarrhea, ReSoMal if patient has SAM  Isotonic fluid bolus used for intravascular volume expansion indicated (severe dehydration or shock) at 20 ml/kg (can be repeated)  10% Dextrose bolus used for management of hypoglycemia at 5 ml/kg | 91%  90%^c^  100% | 63%^22^, 56%^12^, 80%^25^  44%^12^, 30% & 67%^18^  -- |
| Anemia & transfusion | Diagnosis of anemia for hemoglobin <11g/dl^d^  Blood transfusion for hemoglobin ≤4g/dl, hemoglobin >4.0 and ≤5.9g/dl with clinical signs of shock, dehydration, respiratory acidosis, impaired consciousness, heart failure, or severe hyperparasitemia. Persistent shock unresponsive to appropriate intravenous fluid resuscitation is another indication regardless of hemoglobin status. | 19%  66%^c^ | --  66%^22^ |
| Antibiotics | Meningitis: 3^rd^ generation cephalosporin of ampicillin plus gentamicin  Pneumonia: Amoxicillin. Severe Pneumonia: Ampicillin/benzyl penicillin & gentamicin or 3^rd^ generation cephalosporin (second line)  Sepsis: Ampicillin/benzyl penicillin & gentamicin, or 3^rd^ generation cephalosporin  Severe Acute Malnutrition: Ampicillin/ benzyl penicillin & gentamicin^e^  Diarrhea without dysentery, suspected cholera or antibiotic-indicated comorbidity: No antibiotics  Malaria without antibiotic-indicated comorbidity: No antibiotics | 67%  86%  94%  74%  10%  26% | 83%^22^  17-21%^27^, 59%^12^, 50%^23^, 66%^20^, 96%^22^, 92%^21^, 100%^24^  96%^22^  --  19%^23^, 29%^25^, 44%^24^  58%^19^, 19-100%^26^ |
| Malaria diagnosis and antimalarials | Malaria testing for fever^f^  Uncomplicated malaria: Artemether + lumefantrine, artensunate + amodiaquine, artensunate + mefloquine, dihydroartemisinin + piperaquine, artesunae + sulfadoxine-pyrimethamine. Complicated: Intravenous or intramuscular artensunate or artemether | 72%  99% | 91%^12^, 26% & 97%^18^, 97%^26^  74%^12^, 92%^18^, 97%^22^ |
| Screening for and management of severe acute malnutrition | Universal screening by assessment of WHZ, MUAC and edema  Acute phase management: F-75 at 130 ml/kg/day (100ml/kg/day for kwashiorkor) | 52%  78% | ---  25%^12^ |
| HIV testing | Universal screening at African sites, referral for testing if clinically suspected at Asian sites | 38% | 12%^12^, 19% & 23%^18^ |

^a^Derived from WHO Pocket Book of Hospital Care for Children, unless otherwise stated. ^b^89% if those on oxygen during pulse oximetry (regardless if oximetry < or >90%) are included. ^c^Our estimates are based on percent of boluses/transfusions with a documented indication, the comparison estimates were based on the proportion of children with the indication who received the management. ^d^Derived from WHO haemoglobin concentrations for diagnosis of anaemia and assessment of severity. ^e^Gentamicin & chloramphenicol at Queen Elizabeth Central Hospital, per Malawi national guideline. ^f^Among CHAIN sites with high malaria endemicity: Banfora, Kilifi, Migori, Mulago, and Queen Elizabeth Central Hospitals.

Abbreviations: F-75, milk formula 75; kg, kilograms; ml, milliliters; MUAC, mid-upper arm circumference; ORS, oral rehydration solution; WHZ, weight-for-height Z score

Table C. High dependency care (HDC) scores—per hospital unit and across hospital.

| **Site Unit** | **HDC Indicators** | | | | | | | | | |
| --- | --- | --- | --- | --- | --- | --- | --- | --- | --- | --- |
|  | *Dedicated area close to nurse station* | *Dedicated nurse* | *Isolated area* | *Oxygen* | *High flow*  *oxygen* | *CPAP* | *Ventilator* | *Dedicated resuscitation trolley* | *Paediatric intensivist available* | **Total** |
| **Civil** |  |  |  |  |  |  |  |  |  |  |
| *ED* | 1 | 1 | 1 | 1 | 1 | 1 | 0 | 1 | 0 | 7/9 |
| *PICU* | 1 | 1 | 1 | 1 | 1 | 1 | 1 | 1 | 0 | 8/9 |
| *Unit 1* | 1 | 1 | 1 | 1 | 0 | 1 | 0 | 1 | 0 | 6/9 |
| *Unit 2* | 1 | 1 | 1 | 1 | 1 | 1 | 0 | 1 | 0 | 7/9 |
| *Hospital Score* | 1 | 1 | 1 | 1 | 1 | 1 | 1 | 1 | 0 | 8/9 |
| **Dhaka**  *NRU* | 0 | 0 | 0 | 0 | 0 | 0 | 0 | 0 | 0 | 0/9 |
| *LSW* | 0 | 0 | 0 | 1 | 0 | 0 | 0 | 1 | 0 | 2/9 |
| *ICU* | 1 | 1 | 1 | 1 | 1 | 1 | 1 | 1 | 1 | 9/9 |
| *Hospital Score* | 1 | 1 | 1 | 1 | 1 | 1 | 1 | 1 | 1 | 9/9 |
| **Kilifi**  *HDU* | 1 | 1 | 1 | 1 | 1 | 1 | 0 | 1 | 0 | 7/9 |
| *WARD 1* | 1 | 0 | 1 | 1 | 1 | 0 | 0 | 0 | 0 | 4/9 |
| *Hospital Score* | 1 | 1 | 1 | 1 | 1 | 1 | 0 | 1 | 0 | 7/9 |
| **Matlab**  *MCH Ward* | 1 | 0 | 0 | 1 | 0 | 0 | 0 | 1 | 0 | 3/9 |
| *Diarrhoea Ward* | 1 | 0 | 0 | 1 | 0 | 0 | 0 | 1 | 0 | 3/9 |
| *Hospital Score* | 1 | 0 | 0 | 1 | 0 | 0 | 0 | 1 | 0 | 3/9 |
| **Mbagathi**  *Paediatric Ward* | 1 | 0 | 1 | 1 | 0 | 1 | 0 | 1 | 0 | 5/9 |
| *Hospital Score* | 1 | 0 | 1 | 1 | 0 | 1 | 0 | 1 | 0 | 5/9 |
| **Migori**  *Paediatric Ward* | 1 | 0 | 1 | 1 | 0 | 1 | 0 | 0 | 0 | 4/9 |
| *Hospital Score* | 1 | 0 | 1 | 1 | 0 | 1 | 0 | 0 | 0 | 4/9 |
| **Mulago** |  |  |  |  |  |  |  |  |  |  |
| *Acute Care Ward* | 1 | 1 | 1 | 1 | 0 | 1 | 0 | 1 | 0 | 6/9 |
| *PICU* | 1 | 1 | 1 | 1 | 1 | 1 | 0 | 1 | 0 | 7/9 |
| *Malnutrition Ward* | 1 | 0 | 1 | 1 | 0 | 1 | 0 | 1 | 0 | 5/9 |
| *Firms A/C* | 1 | 0 | 0 | 1 | 0 | 1 | 0 | 1 | 0 | 4/9 |
| *Firms B/D* | 1 | 0 | 0 | 1 | 0 | 1 | 0 | 1 | 0 | 4/9 |
| *Hospital Score* | 1 | 1 | 1 | 1 | 1 | 1 | 1 | 1 | 0 | 8/9 |
| **QECH** |  |  |  |  |  |  |  |  |  |  |
| *A&E* | 1 | 1 | 1 | 1 | 0 | 1 | 0 | 1 | 0 | 6/9 |
| *Malnutrition Ward* | 1 | 1 | 1 | 1 | 0 | 1 | 0 | 1 | 0 | 6/9 |
| *Nursery* | 1 | 1 | 1 | 1 | 0 | 1 | 0 | 1 | 0 | 6/9 |
| *Special Care* | 1 | 1 | 1 | 1 | 0 | 1 | 0 | 1 | 0 | 6/9 |
| *Hospital Score* | 1 | 1 | 1 | 1 | 0 | 1 | 1 | 1 | 0 | 7/9 |

Abbreviations: CPAP – Continuous positive airway pressure, A&E – Accident and emergency, ED – Emergency department, HDU – High dependency unit, Hosp. – Hospital, ICU – Intensive care unit, LSW – Long stay ward, NRU. – Nutrition Unit, MCH – Maternal Child Health, PICU – Pediatric intensive care unit, QECH – Queen Elizabeth Central Hospital

Table D. Equipment availability by ward and cumulatively at each hospital.

| **Hospital** | **Civil**  **Pakistan** | | | | | **Dhaka**  **Bangladesh** | | | | **Matlab**  **Bangladesh** | | | **Mulago**  **Uganda** | | | | | | **Mbagathi**  **Kenya** | **Migori**  **Kenya** | **Kilifi**  **Kenya** | | | **QECH**  **Malawi** | | | | |
| --- | --- | --- | --- | --- | --- | --- | --- | --- | --- | --- | --- | --- | --- | --- | --- | --- | --- | --- | --- | --- | --- | --- | --- | --- | --- | --- | --- | --- |
| **Ward** | **Unit 1** | **Unit 2** | **PICU** | **ED** | **Hosp.** | **LSW** | **ICU** | **NRU** | **Hosp.** | **Dia.** | **MCH** | **Hosp.** | **PICU** | **Acute Care** | **Firm A/C** | **Firm B/D** | **NRU.** | **Hosp.** | **Paed**  **Ward &**  **Hosp.** | **Paed Ward &**  **Hosp.** | **HDU** | **Ward 1** | **Hosp.** | **A&E** | **Nutr.** | **Special Care** | **Nursery** | **Hosp.** |
| **Assessment** | 10/11 | 10/11 | 10/11 | 10/11 | **10/11** | 8/11 | 8/11 | 3/11 | **10/11** | 8/11 | 8/11 | **8/11** | 7/11 | 7/11 | 6/11 | 7/11 | 8/11 | **11/11** | **11/11** | **9/11** | 9/11 | 10/11 | **11/11** | 9/11 | 9/11 | 7/11 | 8/11 | **11/11** |
| **Acute Care** | 13/14 | 14/14 | 14/14 | 14/14 | **14/14** | 13/14 | 14/14 | 2/14 | **14/14** | 13/14 | 12/14 | **13/14** | 14/14 | 13/14 | 12/14 | 12/14 | 13/14 | **14/14** | **13/14** | **14/14** | 14/14 | 12/14 | **14/4** | 14/14 | 12/14 | 14/14 | 14/14 | **14/14** |
| **Resuscitation** | 7/7 | 6/7 | 7/7 | 7/7 | **7/7** | 5/7 | 7/7 | 0/7 | **7/7** | 4/7 | 4/7 | **4/7** | 6/7 | 6/7 | 6/7 | 6/7 | 6/7 | **6/7** | **6/7** | **6/7** | 7/7 | 6/7 | **7/7** | 7/7 | 6/7 | 5/7 | 7/7 | **7/7** |
| **Equip. Total** | **29/32** | **29/32** | **31/32** | **31/32** | **31/32** | **16/32** | **30/32** | **5/32** | **31/32** | **25/32** | **24/32** | **25/32** | **27/32** | **26/32** | **25/32** | **25/32** | **27/32** | **31/32** | **30/32** | **28/32** | **30/32** | **29/32** | **32/32** | **29/32** | **27/32** | **26/32** | **29/30** | **32/32** |

Abbreviations: A&E – Accident and emergency, ED – Emergency department, , Equip – Equipment, HDU – High dependency unit, Hosp. – Hospital, ICU – Intensive care unit, LSW – Long stay ward, NRU – Nutrition Unit, Paed – Paediatric, PICU – Pediatric intensive care unit, QECH – Queen Elizabeth Central Hospital
