# Supplementary material for: A mixed method multi-country assessment of barriers to implementing pediatric inpatient care guidelines
Source: PLoS One. 2019 Mar 25;14(3):e0212395. doi: 10.1371/journal.pone.0212395 (PMC6433255; doi:10.1371/journal.pone.0212395)
Supplement: S1 Appendix — (DOCX) [file pone.0212395.s002.docx]

| **Contributions** | **Full Name**  **(Last, First, Middle)** | **Contact information**  **(email, phone number with country code)** | **Affiliations**  **** All are affiliated with the CHAIN Network** |
| --- | --- | --- | --- |
| Network PI | Berkley, JA | JBerkley@kemri-wellcome.org | KEMRI/Wellcome Trust Research Programme, Kilifi, Kenya.  Center for Tropical Medicine and Global Health, University of Oxford, Oxford, United Kingdom. |
| Network PI | Walson, JL | walson@uw.edu | Departments of Global Health, Medicine, Paediatrics and Epidemiology, University of Washington, Seattle, Washington, USA. |
| Site PI/Co-Investigator | Diallo, AH | hama.diallo@univ-ouaga.bf | Department of Public Health, Faculty of Health Sciences, University of Ouagadougou I Pr Joseph KI-ZERBO, Burkina Faso;  Department of Public Health, Centre MURAZ Research Institute, Ministry of Health, Bobo-Dioulasso, Burkina Faso. |
| Co-Investigator | Shahid, ASMSB | sayeem@icddrb.org | Nutrition and Clinical Services Division (NCSD), International Centre for Diarrhoeal Disease Research, Bangladesh (icddr,b), Dhaka, Bangladesh. |
| Co-Investigator | Gwela, A | AGwela@kemri-wellcome.org | KEMRI/Wellcome Trust Research Programme, Kilifi, Kenya |
| Site PI/Co-Investigator | Saleem, A | ali.saleem@aku.edu | Department of Pediatrics and Child Health, The Aga Khan University, Karachi, 74800, Pakistan |
| Co-Investigator | Asad, A | asad.ali@aku.edu | Department of Pediatrics and Child Health, The Aga Khan University, Karachi, 74800, Pakistan |
| Co-Investigator | Tigoi, CC | ctigoi@kemri-wellcome.org | KEMRI/Wellcome Trust Research Programme, Kilifi, Kenya |
| Co-Investigator | Bourdon, C | celine.bourdon@sickkids.ca | The Hospital for Sick Children, Department of Translational Medicine, Toronto, Canada |
| Co-Investigator | Lancioni, CL | lancioni@ohsu.edu | Department of Pediatrics, Oregon Health and Science University, Portland, OR, USA |
| Co-Investigator | Denno, DM | ddenno@uw.edu | Department of Pediatrics, School of Medicine, University of Washington, Seattle, WA. USA.  Department of Global Health and Department of Health Services, School of Public Health, University of Washington, Seattle, WA. USA. |
| Co-Investigator | Mangale, DI | dmangale@uw.edu | Department of Global Health, University of Washington, Seattle, WA. USA. |
| Site PI/Co-Investigator | Mupere, E | mupez@yahoo.com | Department of Paediatrics and Child Health, College of Health Sciences, Makerere University, Kampala, Uganda. |
| Co-Investigator | Tickell, KD | kirkbt@uw.edu | Departments of Global Health, and Epidemiology, University of Washington, Seattle, Washington, USA. |
| Co-Investigator | Mwangome, MK | mmwangome@kemri-wellcome.org | KEMRI/Wellcome Trust Research Programme, Kilifi, Kenya |
| Site PI/Co-Investigator | Chisti, MJ | chisti@icddrb.org | Nutrition and Clinical Services Division (NCSD), International Centre for Diarrhoeal Disease Research, Bangladesh (icddr,b), Dhaka, Bangladesh. |
| Co-Investigator | Ngari, MM | MNgari@kemri-wellcome.org | KEMRI/Wellcome Trust Research Programme, Kilifi, Kenya. |
| Co-Investigator | Ngao, NM | nngao@kemri-wellcome.org | KEMRI/Wellcome Trust Research Programme, Kilifi, Kenya |
| Co-Investigator | Sukhtankar, P | psukhtankar@kemri-wellcome.org | KEMRI/Wellcome Trust Research Programme, Kilifi, Kenya |
| Site PI/Co-Investigator | Bandsma, RHJ | robert.bandsma@sickkids.ca | Centre for Global Child Health, Hospital for Sick Children, Toronto, Ontario Canada.  Division of Gastroenterology, Hepatology and Nutrition, Hospital for Sick Children, Toronto, Ontario Canada.  Department of Nutritional Sciences, Faculty of Medicine, University of Toronto, Toronto, Ontario Canada.  Department of Biomedical Sciences, College of Medicine, University of Malawi, Blantyre, Malawi. |
| Co-Investigator | Molyneux, S | smolyneux@kemri-wellcome.org | KEMRI Centre for Geographic Medicine Research - Coast, and Wellcome Trust Research Programme, Nairobi, Kenya.  Centre for Tropical Medicine, University of Oxford, Oxford, UK. |
| Site PI/Co-Investigator | Ahmed, T | tahmeed@icddrb.org | Nutrition and Clinical Services Division (NCSD), International Centre for Diarrhoeal Disease Research, Bangladesh (icddr,b), Dhaka, Bangladesh. |
| Site PI/Co-Investigator | Voskuijl, W | wp@voskuijl.com | Department of Paediatrics and Child Health, College of Medicine, University of Malawi, Blantyre, Malawi.  Department of Biomedical Sciences, College of Medicine, University of Malawi, Blantyre, Malawi. |
